# Effect of postural insoles on static and functional balance in children with cerebral palsy: A randomized controlled study

**DOI:** 10.1590/bjpt-rbf.2014.0072

**Published:** 2015

**Authors:** Thaluanna C. L. Christovão, Hugo Pasini, Luanda A. C. Grecco, Luiz A. B. Ferreira, Natália A. C. Duarte, Cláudia S. Oliveira

**Affiliations:** Programa de Pós-graduação em Ciências da Reabilitação, Laboratório de Análise do Movimento, Universidade Nove de Julho (UNINOVE), São Paulo, SP, Brazil

**Keywords:** cerebral palsy, balance, orthoses, postural insole, rehabilitation

## Abstract

**BACKGROUND:**

Improved gait efficiency is one of the goals of therapy for children with cerebral palsy (CP). Postural insoles can allow more efficient gait by improving biomechanical alignment.

**OBJECTIVE:**

The aim of the present study was to determine the effect of the combination of postural insoles and ankle-foot orthoses on static and functional balance in children with CP.

**METHOD:**

A randomized, controlled, double-blind, clinical trial. After meeting legal requirements and the eligibility criteria, 20 children between four and 12 years of age were randomly allocated either to the control group (CG) (n=10) or the experimental group (EG) (n=10). The CG used placebo insoles and the EG used postural insoles. The Berg Balance Scale, Timed Up-and-Go Test, Six-Minute Walk Test, and Gross Motor Function Measure-88 were used to assess balance as well as the determination of oscillations from the center of pressure in the anteroposterior and mediolateral directions with eyes open and closed. Three evaluations were carried out: 1) immediately following placement of the insoles; 2) after three months of insole use; and 3) one month after suspending insole use.

**RESULTS:**

The EG achieved significantly better results in comparison to the CG on the Timed Up-and-Go Test as well as body sway in the anteroposterior and mediolateral directions.

**CONCLUSION:**

Postural insoles led to an improvement in static balance among children with cerebral palsy, as demonstrated by the reduction in body sway in the anteroposterior and mediolateral directions. Postural insole use also led to a better performance on the Timed Up-and-Go Test.

## Introduction

The term cerebral palsy (CP) refers to a group of postural and movement disorders stemming from a permanent, non-progressive brain lesion during the development of the immature brain that causes limitations to activities of daily living^1^^,^ 2. Motor impairment is the main manifestation in children with CP, with consequent alterations in the biomechanics of the body^3^^-^ 6. Such children experience a series of limitations due to postural instability during the execution of static and dynamic tasks, such as sitting, standing, and walking^7^^-^ 10.

Postural control and stability are fundamental to motor development. Maintaining the center of gravity of the body over the support base is a complex skill. The clinical state of CP includes neuromuscular deficits, such as the loss of selective motor control and changes in muscle tone, leading to an imbalance between agonist and antagonist muscles, compromised coordination, sensory abnormalities, and muscle weakness^11^. Different therapeutic interventions have been employed in an attempt to improve muscle control and selective coordination in children with CP. Ankle-foot orthoses are normally recommended to control muscle weakness, spasticity, and structural stability of the lower limbs, thereby improving gait quality, postural control, and postural stability^12^^,^ 13.

Studies are currently underway to determine whether postural insoles can produce similar effects to those achieved with ankle-foot orthoses. The findings suggest that postural insoles are capable of reorganizing the tonus of muscle chains and exerting an influence on postural control through correction reflexes, thereby acting on muscle proprioception and resulting in changes in the ascending proprioceptive chains^14^. The stimulation of specific regions of the soles of the feet causes a change in postural tone as well as the repositioning of the pelvis level and muscle asymmetries throughout the spinal column. Postural reprogramming occurs when mechanoreceptors in the plantar region are activated by a deformation of the skin caused by bars, shims, wedges, and half-moon components incorporated into postural insoles^15^. Braces are used to favor function and prevent deformities, but offer some disadvantages, such as limited function, embarrassment on the part of the user due to the visibility of the braces and a high cost. In contrast, postural insoles offer no functional limitations and may favor one's performance in terms of balance. Moreover, postural insoles are approximately 80% less expensive than braces and cause little or no embarrassment.

The aim of the present study was to determine the effect of the combination of postural insoles and ankle-foot orthoses on static and functional balance in children with CP.

## Method

**Figure 1:**
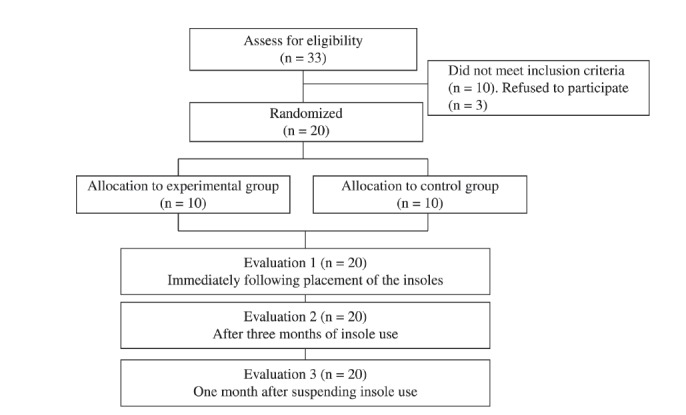
Flowchart of study (CONSORT 2010).

This study received approval from the Human Research Ethics Committee of Universidade Nove de Juho (UNINOVE), São Paulo, SP, Brazil under protocol number 436960 and was carried out in compliance with Resolution 196/96 of the Brazilian National Board of Health. The study is registered with Registro Brasileiro de ensaios clínicos - REBEC under process number RBR6d342s (ensaiosclinicos.gov.br). The legal guardians agreed to the participation of the children by signing a statement of informed consent. A prospective, analytical, randomized, controlled, double-blind, clinical trial was conducted in the Movement Analysis Laboratory, UNINOVE (Figure 1).

Individuals with CP were recruited from the physical therapy clinics of UNINOVE, Brazil. There were no important changes to the methods after trial commencement. Among the 33 children recruited for the present study, ten did not fulfill the eligibility criteria and three refused to participate. Thus, the sample was made up of 20 children with CP, ten of whom were randomly allocated to the control group (CG) and ten to the experimental group (EG).

The inclusion criteria were a diagnosis of spastic diplegic CP and classification as levels of I or II of the Gross Motor Function Classification System (GMFCS)^4^. All children included in the study made use of ankle-foot orthoses and this use was respected. However, the children began to use the insoles in their shoes for a period of six hours per day. Thus, a combination of ankle-foot orthoses and insoles (postural or placebo) was used.

The exclusion criteria were as follows: surgical procedures or the administration of phenol in the previous 12 months; neurolytic block in the previous six months; cognitive or visual impairment that could have interfered with the performance of the procedures; and ankle deformities that were non-reducible to neutral.

### Sample size

The primary outcome was oscillations in the center of pressure (COP). As no previous studies were found on the effects of proprioceptive postural insoles on body sway, the sample size was calculated based on a study in which the effect of ankle-foot orthoses on body sway was investigated. Roque et al.^16^ found a 7.4-mm reduction in mediolateral body sway (expected effect size) in children with CP using ankle-foot orthoses. Considering a standard deviation of 4.7 mm reported in the study, a bi-directional alpha of 0.05 and an 80% test power, a minimum of eight children was determined for each group. The sample was increased by 20% to compensate for possible dropouts, leading to a total of ten children in each group. Thus, the overall sample was made up of 20 male and female children with CP aged four to 12 years.

### Randomization

The participants were randomly allocated to the different groups using cards enclosed in sequentially numbered opaque envelopes. This process was carried out by a member of the team who was not involved in the recruitment process or development of the study. The control group (CG) wore insoles without corrective elements (placebo insoles) and the experimental group (EG) wore insoles with corrective elements (postural insoles). Thus, the conditions for a blind study for the placebo effect of the insole in the CG were satisfied. The examiners and guardians were unaware of the differences between the two types of insole and were blinded to the allocation of the children to the different groups.

### Evaluations

The following standardized assessment tools were used for the evaluation of balance and functional mobility at baseline and after three months of insole use: Berg Balance Scale^17^^-^ 19, Timed Up-and-Go Test^20^^,^ 21, Six-Minute Walk Test^22^^,^ 23, and Gross Motor Function Measure-88 (GMFM-88)^24^. Three evaluations were carried out: 1) immediately following placement of the insoles; 2) after three months of insole use; and 3) one month after suspending insole use.

The Berg Balance Scale (BBS) consists of 14 items that simulate activities of daily living. Each item receives a score ranging from zero (inability to perform task without assistance) to 4 (ability to perform task independently). The total ranges from 0 to 56 points, with higher scores denoting greater independence^17^^-^ 19.

The Timed Up-and-Go Test (TUG) quantifies functional mobility by the time (in seconds) required for a child to stand up from a standardized chair with arm rests, walk three meters, turn around, return to the chair, and sit down again^20^^,^ 21.

The Six-Minute Walk Test is a reliable measure for the assessment of physical fitness and functional mobility that quantifies the distance (in meters) an individual travels in six minutes. This test was performed based on the recommendations established by the American Thoracic Society^11^^,^ 23.

The GMFM-88 is a measure used to quantify gross motor function in individuals with CP. The test consists of observational measures that evaluate motor function through items distributed among six dimensions: A) lying down and rolling; B) sitting; C) crawling and kneeling; D) standing; and E) walking, running, and jumping. The items in each dimension receive a score of 0 to 3 points, with higher scores denoting better performance^24^.

Static balance was evaluated using a force plate (Kistler, model 9286BA), which allows stabilometric analysis through the recording of oscillations in the COP. The sampling frequency was 50 Hz captured by four piezoelectric sensors located at the extremities of the platform (400 × 600 mm). The data were recorded and interpreted using the SWAY program (BTS Engineering) and all data were processed using the SMART-D program (BTS Engineering). This system constitutes a board that integrates the capture and analysis programs regarding kinetic data (used for gait and balance), kinematic data, and electromyographic data. Thus, all the data are synchronized. For the evaluation of static balance, the child was instructed to remain in a quiet standing position on the platform with arms alongside the body, gaze fixed on a point marked at a distance of one meter at the height of the glabellum, with an unrestricted foot base, and heels in alignment. The foot base was unrestricted because the distance between the feet was not standardized based on height, age, and foot size. Readings were made under three conditions: 1) barefoot; 2) in shoes without insoles; and 3) in shoes with insoles (postural insoles with corrective elements in the EG or placebo insoles without corrective elements in the CG). Under all three conditions, the children were evaluated with eyes open and eyes closed.

Evaluations took place on two occasions: immediately after placement of the insoles and after three months of insole use. The order of the different conditions was determined randomly to avoid normalization of the behavior of the sample. Under each condition, displacement from the COP was determined on the x (anteroposterior) and y (mediolateral) axes.

### Intervention

**Figure 2:**
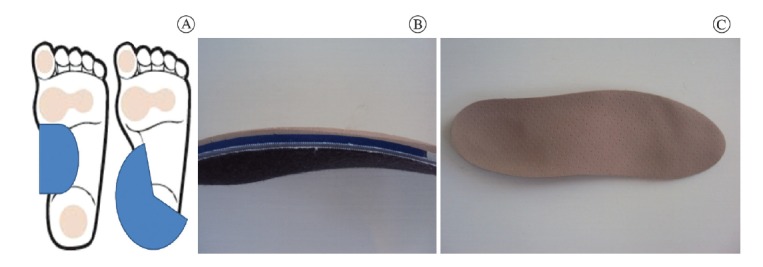
Representation of postural insole; 2A: Position of half-moon and anti-valgus elements; 2B: Side view of three layers of insole after thermal molding; 2C: Top view of insole after thermal molding.

The insoles were prepared by a physical therapist in the city of Sorocaba, SP, Brazil, who used the base insoles and corrective elements fabricated by Podaly(r) (Podaly Posturologia, Brusque, SC, Brazil)*.* Postural insoles are composed of three layers. The aim of the surface layer is to absorb perspiration and provide comfort. The middle portion is made up of ethylene-vinyl acetate measuring 3 mm in thickness. The lower portion is made up of material formed by cotton fibers and resin measuring 1 mm in thickness and contains shims and wedges made of ethylene-vinyl acetate^14^. Half-moon and anti-valgus elements were used in the present study (Figure 2A). After the adequate positioning of the corrective elements, the insoles were thermal molded for the fusion of the different parts (Figure 2B).

### Statistical analysis

The Kolmogorov-Smirnov test demonstrated normal data distribution. Thus, parametric tests were performed, and the data were expressed as mean and standard deviation. Two-way ANOVA with the Bonferroni post-hoc test was used to compare the effects of insole use on functional mobility (Six-Minute Walk Test). The dependent variable was the distance travelled on the test and the fixed independent variables were insole (barefoot and insole), group (EG and CG), and group-treatment interactions. Similar models were run for the other variables. The effect size was calculated considering the mean difference between evaluation times (barefoot before vs. barefoot after; insole before vs. insole after) and groups (EG vs. CG). A p-value <0.05 indicated a statistically significant result. The data were organized and tabulated using the Statistical Package for Social Sciences (v.19.0).

## Results

Table 1 displays the anthropometric characteristics and functional class of the participants. No statistically significant differences between groups were found for the anthropometric data.

**Table 1:** Anthropometric characteristics and functional classification of participants.

	Group	
	Experimentaln=10	Controln=10
Sex (female/male)^*^	8/2	7/3
GMFCS (I/II)^*^	7/3	7/3
Age (years)^**^	7.1 (4-9.7)	6.8 (5-9)
Body mass (kg)^**^	25 (22-26)	24.8 (21-29)
Height (cm)^**^	120.7 (112-128.7)	116.2 (108-122)

* Frequency (n) of children in each group;

** Mean and 95% confidence interval.

The inter-group analysis demonstrated no significant differences immediately following the placement of the postural insoles with corrective elements in the EG and placebo insoles without corrective elements in the CG, with the exception of the shorter time required to execute the TUGT in the EG. In the intra-group analysis, no significant differences were found in either group immediately after placement of the insoles.

During the evaluations conducted after three months of insole use, no significant differences were found in any of the variables in either group in the comparison of the barefoot and shoes + insoles conditions, demonstrating that the performance of the children was similar with and without insoles after three months of insole use for six hours per day. In the intra-group analysis, the EG demonstrated significant reductions in the time required to execute the TUGT as well as in body sway in the anteroposterior and mediolateral directions with eyes open. No significant differences in any other variables were found in the EG and no differences were found in any of the variables in the CG after three months of insole use.

When intragroup analysis was performed considering the three conditions (barefoot, shoes without insoles, and shoes with insoles), significant differences were only found in the EG group on the TUGT (shoes with insoles vs. shoes without insoles, p=0.021) and anteroposterior sway with eyes closed (shoes without insoles vs. barefoot, p=0.050) (Table 2).

**Table 2:** Results after three months of insole use in two groups classified as barefoot and shoes + insoles.

	Before		After	
	Barefoot	Insoles	Barefoot	Insoles
6-minute walk test (m)				
Experimental group	346.0(85.9)	340.6(88.0)	378.8(75.0)	385.1(77.7)
Control group	338.4(87.4)	345.5(74.4)	347.0(69.7)	361.1(7.1)
Timed up-and-go test(s)				
Experimental group	11.7(2.3)	11.9(2.6)	9.7(2.3)*#	9.9(2.0)^*#^
Control group	11.9(2.0)	10.5(2.1)	11.7(2.2)	11.8(0.8)
Balance Berg Scale				
Experimental group	48.6(4.2)	48.6(3.3)	50.6(3.4)	50.7(3.6)
Control group	50.7(3.5)	49.3(3.7)	50.9(3.8)	50.1(3.9)
GMFM-88				
Experimental group	46.4(1.0)	46.9(1.2)	46.9(1.0)	47.1(1.1)
Control group	46.1(1.1)	46.0(1.1)	46.3(0.9)	46.5(0.7)
Anteroposterior eyes open(mm)				
Experimental group	23.5(6.4)	21.3(4.5)	17.4(5.0)^*#^	11.1(5.2)^*#^
Control group	20.1(7.4)	19.9(8.2)	18.7(1.9)	18.3(1.9)
Anteroposterior eyes closed (mm)				
Experimental group	27.3(2.1)	26.9(3.4)	25.3(1.1)	24.9(5.8)
Control group	26.3(5.1)	27.4(7.2)	27.3(9.2)	27.1(7.3)
Mediolateral eyes open (mm)				
Experimental group	30.4(1.0)	31.3(5.2)	25.4(1.9)^*#^	24.7(5.8)^*#^
Control group	32.9(8.9)	31.6(7.8)	31.9(4.7)	31.8(7.6)
Mediolateral eyes closed (mm)				
Experimental group	36.7(6.4)	39.9(10.5)	37.5(9.5)	36.9(5.6)
Control group	35.9(3.5)	37.8(2.9)	37.9(9.5)	36.5(2.6)

* ANOVA, p<0.05 (experimental group after different from before);

# ANOVA, p<0.05 (experimental group different from control group).

## Discussion

Changes in balance and functional mobility are important to the rehabilitation of children with CP. The present findings suggest that postural insoles lead to improvements in static balance and mobility and contribute toward a better understanding and integration of therapeutic resources employed in physical rehabilitation applied to pediatric neurology.

Children with CP exhibit delayed development in terms of balance and stability in comparison to normal children. According to Liao and Hwang^25^, the slower gait velocity and greater physiological cost of walking contribute to poorer static and dynamic balance in comparison to children without disabilities. However, Shumway-Cook et al.^26^ state that children with CP are capable of change through the application of stimuli (even after pre-adolescence) and can reach adult reference standards. The present findings also suggest the possibility of changes in response to stimuli, especially in the long term, as no immediate effects of postural insole use were found.

There are no previous studies on improvements in balance and functional mobility in patients with CP following the use of proprioceptive insoles. However, a number of studies have demonstrated the benefits of such insoles in terms of other aspects. Palluel et al.^27^ evaluated the effects of spiked insoles on balance and found no significant changes immediately following placement, but reported a significant improvement in postural balance after five minutes of insole use. According to Gross et al.^28^, proprioceptive insoles demonstrate immediate effects on static and dynamic balance, which are maintained over time. Pasini^29^ report an immediate improvement in gait velocity and cadence among children with CP classified as GMFCS levels I and II with the use of postural insoles. Likewise, the present study demonstrates that such insoles constitute an important tool for improving functional balance in children classified as GMFCS levels I and II.

The present findings suggest that postural insoles cause a change in sensorial afference, stimulating a postural reaction that favors a better biomechanical alignment of the body and permits more efficient function, especially with regard to balance. One of the hypotheses for this improvement is supported by the findings described by Ceci et al.^30^ and Przysiezny et al.^31^: changes in postural tone occur when mechanoreceptors in specific regions of the soles of the feet are stimulated.

Gan et al.^32^ state that balance is the most important aspect of motor skills. In a review of the literature, Uzun^33^ concluded that the Berg Balance Scale is often employed for the evaluation of this variable and allows a quantitative analysis of the evolution of general motor functions in children with CP based on improvements in balance. However, the present findings allow one to infer that specific changes in static balance may not exert a significant influence on functional equilibrium measured using the Berg Balance Scale.

Nobre et al.^34^ found less oscillation in the anteroposterior direction in children with CP with eyes open and closed. In the present study, reductions in body sway in the anteroposterior and mediolateral directions only occurred with eyes open. The present results are in agreement with data reported by Bulla et al.^35^, who found a 50% improvement in body sway in the mediolateral direction and a 30% improvement in the anteroposterior direction in children with CP using postural insoles.

## Conclusion

Postural insoles led to an improvement in static balance among children with cerebral palsy, as demonstrated by the reduction in body sway in the anteroposterior and mediolateral directions. The use of postural insoles also led to improved performance on the Timed Up-and-Go Test.
